# Huiyang Shengji Extract Improve Chronic Nonhealing Cutaneous through the TGF-*β*1/Smad3 Signaling Pathway

**DOI:** 10.1155/2021/8881565

**Published:** 2021-06-09

**Authors:** Yan Lin, Xiujuan He, Xinran Xie, Qingwu Liu, Jia Chen, Ping Li

**Affiliations:** ^1^Beijing Institute of TCM, Beijing Hospital of Traditional Chinese Medicine, Capital Medical University, 23 Gallery Backstreet, Dongcheng District, Beijing 100010, China; ^2^Beijing University of Chinese Medicine, No. 11, North Third Ring Road, Chaoyang District, Beijing 100029, China

## Abstract

Chronic nonhealing cutaneous wounds are a thorny problem in the field of surgery because of their prolonged and unhealed characteristics. Huiyang Shengji extract (HSE) is an extract of traditional Chinese medicine prescription for treating chronic wounds. This study aims to investigate the regulation of M1 macrophages on fibroblast proliferation and secretion and the intervention mechanism of Huiyang Shengji extract. We found that the effects of HSFs stimulated with paracrine factors from M1 macrophages were as follows: the proliferation of HSFs was reduced, the expression of MKI-67 was downregulated, and the content and gene expression of the inflammation factors and fibroblast MMPs were increased, while the content and gene expression of TIMP-1 are decreased, the content of human fibroblasts secreting type I collagen (COL1A1) and type III collagen (COL3A1) was decreased, and the TGF-*β*1/Smad3 signaling pathway was inhibited. Interestingly, HSE inhibited these effects of M1 macrophages on human fibroblasts after the intervention, and the inhibitory effect was related to the concentration. In conclusion, M1 macrophages caused changes in HSFs and secretion, while HSE has a specific regulatory effect on the proliferation and secretion of fibroblasts caused by M1 macrophages.

## 1. Introduction

In wound healing and fibrosis, various processes are crucial, such as inflammation, cell proliferation, cell migration, and extracellular matrix (ECM) remodeling. Macrophages and fibroblasts play an important role in this process. Fibroblasts are the most abundant cells in connective tissue and one of the critical elements in wound healing. It promotes the generation and reconstruction of the ECM by differentiating into myofibroblasts to restore the physical integrity of connective tissue [1, 2]. Macrophages, as a critical factor in wound healing, are strictly related to the function of fibroblasts. Since macrophages have an M1-proinflammatory phenotype and M2-repair phenotype, the regulation of macrophages on fibroblasts has a dual role. Ploeger et al. [3] found that inflammatory factors secreted by M1 macrophages increased matrix metalloproteinase 2 (MMP-2) and MMP-9 in fibroblasts and upregulated the expression of proinflammatory factors, such as chemokine (C-C motif) ligand 2 (CCL2) (monocyte chemotactic protein-1), CCL7 (monocyte chemotactic protein-3), and interleukin 6 (IL-6), causing fibroblast proinflammatory phenotype and ECM degradation. S100A8/A9 induces the activation of the NF-*κ*B signaling pathway and MMP-9 protein expression in human heart fibroblasts by promoting the secretion of the tumor necrosis factor-*α* (TNF-*α*) by macrophages [4]. M2 macrophages can promote the myofibroblastic activity of human fibroblasts by promoting cell proliferation, increasing collagen content, improving *α*-smooth muscle actin (*α*-SMA), and promoting fibrosis gene expression and the concentration of M2 macrophage-related factors. At the same time, M2 macrophages can reduce the related gene expression of antifibrosis by inhibiting the activity of MMP-1 [5].

Transforming growth factor-*β*1 (TGF-*β*1) plays a vital role in the transformation of fibroblast phenotype, mainly through the TGF-*β*1/Smad signaling pathway to regulate the transformation of fibroblasts to myofibroblasts [6]. TGF-*β*1 transfers extracellular signals to the cytoplasmic effector molecule Smad3 protein through the type II and type I receptors on the cell membrane. The activated Smad3 then enters the nucleus and the target genes, such as ECM binding that regulate the transcription of these target genes [7]. Smads can mediate the transcription activity of transcription factor AP-1 (dimer of c-Fos and c-Jun) via TGF-*β*1, and their binding sites overlap each other. After TGF-*β*1 induces the activation of Smads, Smad3/Smad4 can bind to JunB, c-Jun, June, and c-Fos; c-Jun and Smad3 can simultaneously bind to the overlapping sequence on the TGF-*β*1, and the aminoterminal structure of c-Fos changes may allow the Smads/AP-1 complex to interact with an AP-1 site, which may be an essential site for cross-linking of the Smads signaling pathway and the mitogen-activated protein kinase (MAPK)/Jun N-terminal Kinase (JNK) signaling pathway [8–10]. After the transformation of M1 macrophages into M2 macrophages, the TGF-*β*1 secreted by the cells is significantly increased. The increased TGF-*β*1 can promote the transformation of fibroblasts to myofibroblasts, thereby increasing the synthesis of the extracellular matrix and cell contraction function to promote wound healing [11].

In chronic wounds, macrophages and fibroblasts have changes in morphology and function, which may be related to the involvement of inflammatory mediators locally gathered on the wound surface and the cell apoptosis in the delayed wound healing process [12]. Loots et al. [13] found that the fibroblasts of diabetic ulcers have abnormal morphology and reduced proliferation capacity. It was found that the production of fibroblast MMPs increased in diabetic patients' skin, which is a significant factor leading to the poor healing of diabetic foot ulcers. Rohl and Murray [14] reported that the fibroblast release of MMPs (MMP-2 and MMP-9) increased, and the concentration of matrix metalloproteinase inhibitor 1 (TIMP-1) decreased in chronic wounds compared with normal wounds, which may be related to the continuous inflammatory state of wounds. Sindrilaru et al. [15] demonstrated that many M1 macrophages and proinflammatory factors in human and animal wounds are related to wound healing disorders. Studies have shown that the phenotype of inflammatory (M1 type) to repair (M2 type) phenotypes of wound macrophages in db/db mice is one reason for their slow healing [16]. Thus, in chronic wounds, macrophage phenotype transformation dysfunction, inflammatory macrophages (M1 type) persist, and its anti-inflammatory and prorepair functions are difficult to exert, and a large number of inflammatory mediators are secreted; these factors make fibroblast proliferation and secretion dysfunction, which prevents wound repair from being carried out in time, leading to obstacles to wound healing. Huiyang Shengji ointment exhibits a satisfactory clinical effect in patients with the nonhealing wound and has been produced and applied in Beijing Hospital of TCM for sixty years [17]. Huiyang Shengji ointment is mainly used to treat chronic skin ulcers with Yin syndrome (traditional Chinese medicine). After treatment, the color of the sore surface changed from dull to ruddy, the exudate changed from thin to thick, the shape of the sore changed from flat to granulation and epidermal growth, and the wound changed from an inflammatory state to a repaired state. The traditional clinical technique of Huiyang Shengji ointment is to mix the water extract of decoction pieces and Vaseline. In our previous research, we found that the alcohol extract of Huiyang Shengji decoction was better than the water extract on the proliferation of macrophages and the promotion of macrophage phagocytosis. Therefore, this article selected the alcohol extract of Huiyang Shengji recipe powders for research. Optimizing the process of Huiyang Shengji ointment will provide a theoretical basis in the future.

Our previous study showed that the extract of Huiyang Shengji extract (HSE) affects promoting the proliferation of fibroblasts in vitro [18]. However, the proliferative effect is relatively weak compared with the proliferative effect of the growth factor preparations already in clinic, which is not synchronized with the clinical efficacy of HSE, suggesting that the role of HSE is not only reflected in the role of fibroblasts, it may have a specific regulatory effect on the proliferation and secretion of fibroblasts by M1 macrophages, thereby promoting wound healing.

M1 macrophages can affect the proliferation and secretion of fibroblasts and inhibit the transformation of fibroblasts into myofibroblasts. The TGF-*β*1/Smad signaling pathway plays a key role [19]. Therefore, in this study, we cocultured the supernatant of M1-type macrophages containing different HSE concentrations with fibroblasts to simulate the wound microenvironment and observed the proliferation of fibroblasts, inflammatory factors, MMPs and TIMPs, collagen secretion, and gene expression to study whether HSE inhibited the stimulating effect of M1 macrophages on fibroblasts through the TGF-*β*1/Smad signaling pathway.

## 2. Materials and Methods

### 2.1. Drug Administration

Huiyang Shengji recipe, a Chinese herbal compound, is composed of Cinnamomi cortex (*Cinnamomum cassia* Presl cortex), Ginseng radix et rhizome (*Panax ginseng* C.A.Mey., root and rhizome), Chuanxiong rhizoma (*Ligusticum chuanxiong* Hort., rhizome), and Cervi cornu pantotrichum (*Cervus nippon* Temminck or *Cervus elaphus* Linnaeus, young cornu) (Editorial Committee of Pharmacopoeia of Ministry of Health P. R. China, 2010). Huiyang Shengji recipe powders were provided by Beijing Hospital of TCM, affiliated with Capital Medical University (Beijing, China). The powders were immersed in 95% alcohol, followed by flash extraction with an herbal blitzkrieg extractor (JHBE-50T, Henan Jinnai Tech. Co., Ltd.) and centrifugation at 4000 rpm for 10 minutes. Then, supernatant was decompressed and recycled to evaporate it to powder. The powder was resolved in 1640 medium supplemented with 10% fetal bovine serum (FBS) for cells intervention. The alcohol extract of HSE was used for in vitro experiments. The chemical profile of HSE was established by ultraperformance liquid chromatography coupled with quadrupole time-of-flight mass spectrometry (UPLC/Q-TOF-MSE) analysis. The results implied that phenolic acids, lactones, and saponins, including ferulic acid, Ligustrum, cinnamaldehyde, cis-trans-Ligustrum, esters, ligustilide A, n-butylphthalide, phthalide dimers, lysolecithin, and rosin acid [18, 20].

### 2.2. Macrophage Cell Culture, Polarization with M1 Stimuli, and Collection of Conditioned Media

Human monocytic cell line THP-1 was obtained from the National Experimental Cell Resource Sharing platform (Beijing, China) and 5-8 generations used in the study. THP-1 was maintained at 2 × 10^5^ cells/ml in RPMI 1640 GlutaMAX™ medium supplemented with 10% FBS and 2 mmol/L L-glutamine (Gibco BRL). THP-1 cells (2 × 10^5^/ml) were differentiated to M0 macrophages using 100 ng/mL PMA (phorbol 12-myristate 13-acetate, Sigma-Aldrich, St.Louis, USA) for 24 h. The adherent cells (M0 macrophages) were washed and stimulated in culture medium with 100 ng/mL LPS (Sigma-Aldrich, St.Louis, USA) + 40 ng/ml IFN-*γ* (Sigma-Aldrich, St. Louis, USA) at 37°C for 48 h. The polarization state of the macrophages was determined by quantitative RT-PCR (qRT-PCR). The cells were subsequently washed and cultured in DMEM medium with either the HSE-L group with 0.064 mg·L^−1^ HSE, the HSE-M group with 0.32 mg·L^−1^ HSE, the HSE-H group with 1.6 mg·L^−1^ HSE, and no medicine (model) for 24 h with 5% CO_2_ at 37°C. After 4 h, CM from the HSE-L group, HSE-M group, HSE-H group, and model group macrophages were collected and stored for further analyses at −20°C. The CM of the different conditions was used for stimulation of HSF.

### 2.3. HSFs Culture and Stimulation with M1 Conditioned Medium (CM) of Different Conditions

Human skin fibroblasts cells (HSFs) were obtained from the National Experimental Cell Resource Sharing platform (Beijing, China), and 5–9 generations were used in the study. HSFs were seeded into culture plates overnight with a density of 5 × 10^4^ cells/cm^2^ in DMEM medium containing 1% penicillin/streptomycin and 20% FBS with 5% CO_2_ at 37°C. The next day, HSFs were washed and cultured in DMEM medium with either. After 24 h, the DMEM medium was replaced by CM derived of M1 or stimulated M1 with HSE-L, HSE-M, and HSE-H for 24 h or 48 h at 37°C.

### 2.4. Cell Viability Assay

According to the manufacturer's protocol, cell viability was determined using the the Cell Counting kit-8 (Dojindo Molecular Technologies, Inc., Kumamoto, Japan). HSFs were seeded in 96-well plates (1 × 10^5^ cells/ml) with various HSE concentrations for 24 h and 48 h and were then incubated with CCK-8 reagent for 3 h at 37°C. Absorbance was measured at 450 nm and expressed as an arbitrary unit proportional to cell toxicity. For each of these experiments, at least three parallel measurements were performed.

### 2.5. Immunofluorescent Stainings for MKI67-Stimulated HSFs

After 48 h, HSFs seeded into confocal plates were washed twice with PBS (Hyclone, USA). The fixed cells were incubated with formaldehyde: acetone (1 : 1) for 8 min. Then, cells were incubated with rabbit antihuman MKI67 (1 : 500) (Abcam, ab15580, USA) diluted in PBS overnight at 4°C. Cells were incubated with goat-anti-rabbit-FITC (Abcam, ab6717, USA) (1 : 500) diluted in PBS at 37°C for 1 h. Cells were examined by a laser scanning confocal microscope (LS780, ZEISS, Germany) equipped with Application Suite software.

### 2.6. Luminex® Cytokine Detection

CM of stimulated HSFs was collected at 24 h and 48 h, and CCL2, CCL7, IL-6, MMP-1, MMP-2, and MMP-3 secretion by HSFs was determined by the human cytokine Luminex custom 7-plex kits (ThermoFisher, ProcartaPlex, USA) in accordance to manufacturer's protocol. Median fluorescence intensity (MFI) for each sample was registered and analyzed with Bio‐Plex Manager™ Software version 5.0 (BioRad Laboratories). Briefly, cytokine-specific antibodies were precoated on the magnetic microparticles, and 50 *µ*L of sample per well was used to perform the assay. The assays were performed in duplicate. Biotin antibody cocktails specific to the cytokines were added to each well. The prepared microplates were analyzed with the Luminex® 100/200® system (Luminex Corporation, Texas, USA). Quantitative data for each cytokine was derived based on the microparticle regions specified by the manufacturer. Data were acquired as median fluorescence intensity and concentration in picogram/milliliter, and analysis of experimental data was carried out using a five-parametric curve fitting with the use of the xPONENT® software (Luminex Corporation, Texas, USA).

### 2.7. Reverse Transcription-Quantitative PCR (RT-qPCR)

Total RNA was extracted from the cells at 72 h and 144 h using TRIzol® reagent (Thermo Fisher Scientific, Inc.) and purified using the NucleoSpin® RNA Clean-up kit. The following generation of cDNA uses the AffinityScript Multiple Temperature cDNA Synthesis kit according to the manufacturer's protocol. The real-time PCR FastStart Universal Master Mix (Roche Diagnostics) was used to determine the relative expression levels of genes with an ABI 7500 Fast Real-Time PCR system (Applied Biosystems, Thermo Fisher Scientific, Inc.). The reactions were performed at 95°C for 15 s, 60°C for 40 s, and 40 cycles. The gene-specific primers are given in Table 1.

### 2.8. Western Blotting

After 72 h, the HSFs were washed with PBS and lysed in lysis buffer (Cell Signaling Technology, Inc., Danvers, MA, USA) supplemented with proteinase and phosphatase inhibitor cocktails on ice. The lysates were collected after centrifugation at 12,000*g* for 30 minutes at 4°C. The BCA protein quantification kit (ThermoFisher, USA) was used to quantify the total protein of the extracted cells. 20 *μ*g sample protein from each group was subjected to SDS-polyacrylamide gel electrophoresis (SDS-PAGE) and transferred to PVDF membrane and blocked with 5% skimmed milk powder blocking solution at room temperature for 1 h. Then, the membranes were incubated at 4°C overnight with the antibodies for *α*-SMA (Immunoway, USA), Smad3 (Abcam, USA), p-Smad3 (Abcam, USA), c-FOS (Abcam, USA), p-c-FOS (Abcam, USA),c-Jun (Abcam, USA), p-c-Jun (Abcam, USA), and GAPDH (Abcam, USA). After washed with TBS, the membranes were added secondary antibody (1 : 10 000) and shook gently at room temperature for 40 minutes, and ECL was added to the membrane to react for 3–5 minutes, and the film was exposed. The density of the bands was normalized to *β*-actin and quantified with Image software. Immunofluorescence was assessed using an Odyssey infrared imaging system (LI-COR Biosciences, Lincoln, NE, USA).

### 2.9. Statistical Analysis

All experiments were performed three times independently. SPSS 20.0 software was used for statistical analysis of the data. The data were presented as mean ± standard deviation (*x* ± *s*). Those with normal distribution and homogeneous variance were tested by one-way analysis of variance. The LSD test was used for comparison between the two groups, while those without normal distribution or heterogeneous variance were tested by the nonparametric test. *P* < 0.05 was considered statistically significant.

## 3. Results

### 3.1. Confirmation of Differentiation of M1 Macrophage

**Figure 1:**
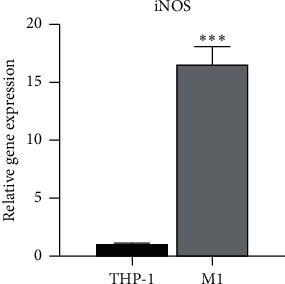
Characterization of macrophages after M1 polarization. The significantly increased expression of iNOS mRNA as the surface marker of M1 macrophage cells indicates the successful differentiation of M1 macrophages. ^*∗∗∗*^*P* < 0.001 compared with the THP-1 group.

RT-PCR was used to detect the gene expression of iNOS, which is the M1 macrophage marker, to identify M1 macrophages. Compared to unstimulated macrophages, macrophages stimulated with PMA/LPS/IFN-ϒ showed an upregulation of the gene expression of iNOS (*P* < 0.01), which indicated the successful differentiation of M1 macrophages (Figure 1).

### 3.2. Effect of HSE on the Proliferation of HSF Stimulation with the M1 CM of Different Conditions

**Figure 2:**
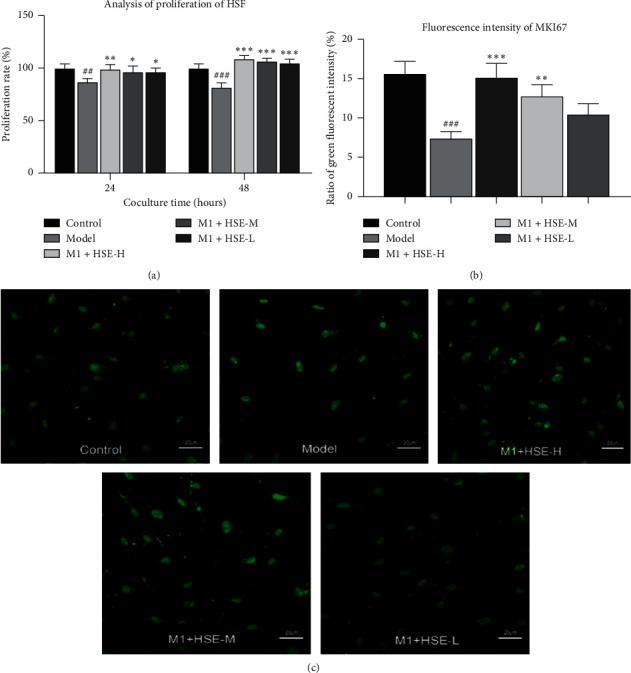
Effect of HSE on the proliferation of HSF cocultured with the CM of M1 macrophages. (a) After cocultivation with the CM of M1 for 24 hours, the number of fibroblasts decreases. Intervention with different concentrations of HSE can promote the proliferation of fibroblasts. After 48 hours, the effect of promoting proliferation is more significant. (b), (c) The expression of MKi-67 in fibroblasts cocultured with the CM of M1 was reduced. After 48 hours, the CM of M1with different concentrations of HSE can promote the expression of MKi-67 in fibroblasts. ^#^*P* < 0.05, ^###^*P* < 0.01, and ^###^*P* < 0.001 compared with the control group; ^*∗*^*P* < 0.05, ^*∗∗*^*P* < 0.01, and ^∗∗∗^(P) < 0.001 compared with the model group.

The CM of M1 inhibited the proliferation of fibroblasts. After 24 h and 48 h, the proliferation of fibroblasts was significantly reduced compared with the model group. After 48 h, the expression of MKI-67 in fibroblasts was significantly reduced (*P* < 0.05). However, the M1 CM of HSE had no inhibitory effect on the proliferation of fibroblasts. Compared with the model group, they could promote the proliferation of fibroblasts. After 24 h, the HSE-H group had the most substantial effect (*P* < 0.01). There was also a significant difference between the HSE-M group and the HSE-L group (*P* < 0.05). After 48 h, the HSE group promoted fibroblast proliferation more significantly (*P* < 0.01), and MKI-67 expression of HSF increased (*P* < 0.05) (Figure 2).

### 3.3. Effect of HSE on the Secretion of CCL2, CCL7, and IL-6 by HSF Stimulation with the M1 CM of Different Conditions

**Figure 3:**
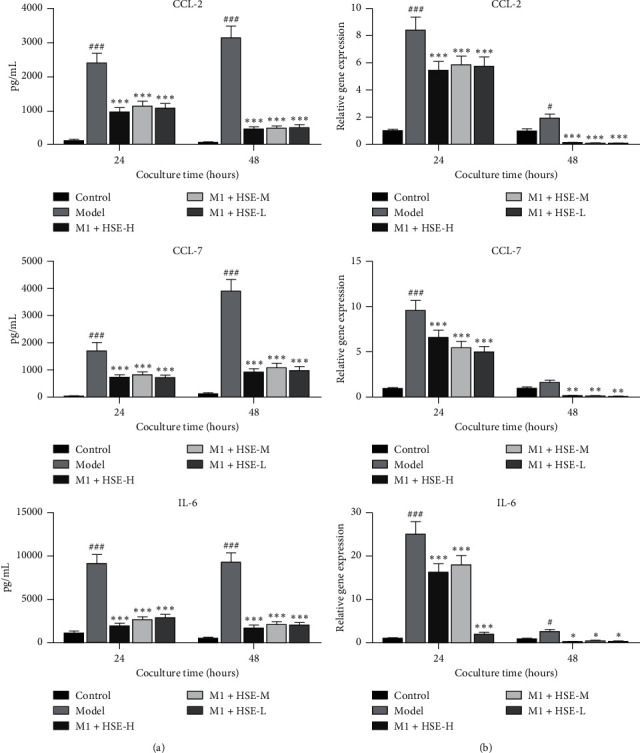
Effect of HSE on the secretion of CCL2, CCL7, and IL-6 by HSF cocultured with the CM of M1 macrophages. (a) The secretion of CCL2, CCL7, and IL-6 by HSF under different intervention conditions. (b) The relative gene expression of CCL2, CCL7, and IL-6 of HSF under different intervention conditions. The data are mean ± standard deviation. ^#^*P* < 0.05, ^###^*P* < 0.01, and ^###^*P* < 0.001 compared with the control group; ^*∗*^*P* < 0.05, ^*∗∗*^*P* < 0.01, and ^*∗∗∗*^*P* < 0.001 compared with the model group.

After stimulation with the M1 CM, the secretion of CCL2, CCL7, and IL-6 by fibroblasts increased (*P* < 0.01). However, M1 CM of different HSE concentrations could inhibit the secretion of CCL2, CCL7, and IL-6 by HSF (*P* < 0.01), and the inhibitory effect of the HSE-H group was the strongest (*P* < 0.05). The effect of inhibiting the secretion of CCL7 by HSF was more substantial after HSE intervention for 24 h, and the inhibition of CCL2 and IL-6 by HSF was more potent after intervention for 48 h (*P* < 0.05) (Figure 3).

### 3.4. Effect of HSE on the Secretion of MMP-1, MMP-2, MMP-3, and TIMP-1 by HSF Stimulation with the M1 CM of Different Conditions

**Figure 4:**
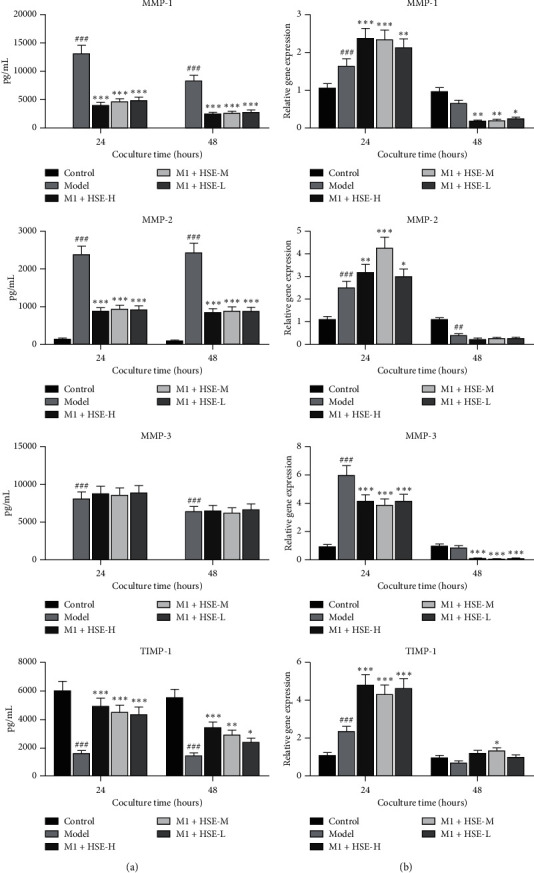
Effect of HSE on the secretion of MMP-1, MMP-2, MMP-3, and TIMP-1 by HSF cocultured with the CM of M1 macrophages. (a) The secretion of MMP-1, MMP-2, MMP-3, and TIMP-1 by HSF under different intervention conditions. (b) The relative gene expression of MMP-1, MMP-2, MMP-3, and TIMP-1 of HSF under different intervention conditions.The data are mean ± standard deviation. ^#^*P* < 0.05, ^###^*P* < 0.01, and ^###^*P* < 0.001 compared with the control group; ^*∗*^*P* < 0.05, ^*∗∗*^*P* < 0.01, and ^*∗∗∗*^*P* < 0.001 compared with the model group.

After stimulation with the M1 CM of different conditions for 24 h and 48 h, HSF secreted more MMP-1, MMP-2, and MMP-3 and secreted less TIMP-1 (*P* < 0.01). After the intervention of different concentrations of HSE, M1 macrophages can inhibit fibroblast secretion of MMP-1, MMP-2, and MMP-3 and promote fibroblast secretion of TIMP-1 (*P* < 0.01). After 24 h of intervention, it had a more substantial promotion effect on TIMP-1, and after 48 h of intervention, it had a more substantial inhibitory effect on MMP-1 and MMP-3, and high concentration of HSE had a more substantial inhibitory effect on MMP-1 and promotion effect on TIMP-1 (*P* < 0.05) (Figure 4).

### 3.5. Effect of HSE on the Secretion of COL1A1 and COL3A1 by HSF Stimulation with the M1 CM of Different Conditions

**Figure 5:**
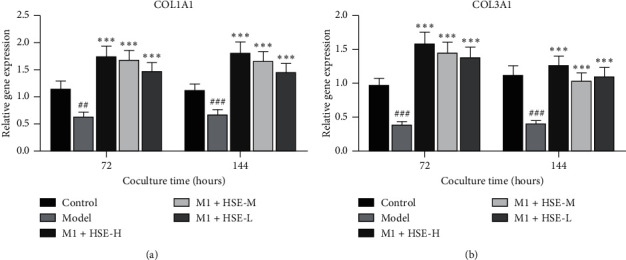
Effect of HSE on the secretion of (a) COL1A1 and (b) COL3A1 by HSF cocultured with the CM of M1 macrophages. The relative gene expression of COL1A1 and COL3A1 of HSF under different intervention conditions. The data are mean ± standard deviation. ^#^*P* < 0.05, ###*P* < 0.01, and ###*P* < 0.001 compared with the control group; ^*∗*^*P* < 0.05, ^*∗∗*^*P* < 0.01, and ^*∗∗∗*^*P* < 0.001 compared with the model group.

After stimulation with the CM of M1, 72 h and 144 h, the secretion of COL1A1 and COL3A1 by HSF decreased. While HSF secreted more COL1A1 and COL3A1 after different concentrations of HSE interfered with M1 macrophages (*P* < 0.01).After 144 hours of intervention, the high concentration of HSE had a strong promoting effect on the secretion of COL3A1 by fibroblasts (*P* < 0.05) (Figure 5).

### 3.6. Effect of HSE on the Expression of Fibroblast Phenotype Transformation Pathway-Related Proteins *α*-SMA, Smad3, c-Fos, and c-Jun

**Figure 6:**
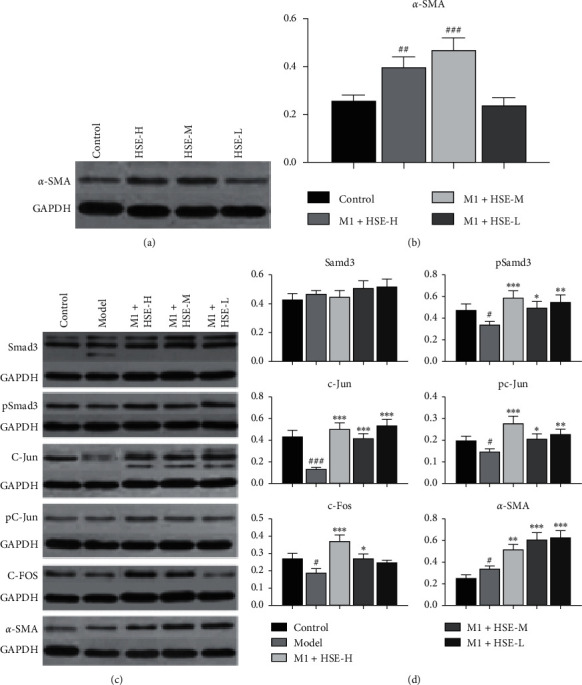
Effect of HSE on the expression of fibroblast phenotype transformation pathway-related proteins *α*-SMA, Smad3, c-Fos, and c-Jun. Western blot analysis of *α*-SMA, Smad3, p-Smad3, c-FOS, p-c-FOS, c-Jun, p-c-Jun, GAPDH. GAPDH was loading control. The relative protein levels were normalized to GAPDH and calculated by densitometry. The data are mean ± standard deviation. ^#^*P* < 0.05, ^###^*P* < 0.01, and ^###^*P* < 0.001 compared with the control group; ^*∗*^*P* < 0.05, ^*∗∗*^*P* < 0.01, and ^*∗∗∗*^*P* < 0.001 compared with the model group.

After 72 h of incubation with HSE in HSF, the *α*-SMA of HSF in the HSE-H and HSE-M groups increased significantly, but there was no significant change in the HSE-L group (Figures 6(a) and 6(b)). After stimulation with the M1 CM of different conditions, the expression levels of TGF-*β*1/Smad3 pathway-related proteins and their phosphorylated proteins in different concentration groups were increased to varying degrees. Furthermore, these protein expression changes were related to the concentration of HSE.

## 4. Discussion

Fibroblasts have high dynamic plasticity in different stages of tissue repair [3]. In this study, we detected the proliferation of fibroblasts, which were cocultured with the superfluid of M1 macrophages, and the results showed that M1 macrophages inhibited the proliferation of fibroblasts at 24 h and 48 h, which was consistent with previous research results [21]. However, after the intervention with HSE for 24 h, the inhibitory effect on the proliferation of fibroblasts almost disappeared, and the number of fibroblasts increased significantly, indicating that HSE can regulate the inhibitory effect of M1 macrophages on the proliferation of fibroblasts. MKI-67 is a nuclear protein essential for cell proliferation and used as a cell proliferation marker [22]. In this study, MKI-67 was used to observe the proliferation status of fibroblasts further. The decrease of MKI-67 expression of fibroblasts stimulation with the CM of M1 also confirmed the decrease of fibroblast proliferation. Similarly, after the HSE intervention, the inhibitory effect of M1 macrophages on fibroblasts was eliminated.

M1 macrophages can promote the fibroblasts to exhibit a proinflammatory phenotype through the paracrine pathway. Previous studies have shown that the coculture of LPS-activated macrophages and fibroblasts increases the production of proinflammatory cytokines and chemokines [23]. It mainly includes CCL2 and CCL3, which are related to the factors secreted by M1 macrophages [24]. In the results of this study, fibroblasts cocultured with M1 macrophages supernatants increased CCL2, CCL7, and IL-6 secretion. After the intervention of HSE in M1 macrophages, the secretion of CCL2, CCL7, and IL-6 by fibroblasts cocultured with the superscript solution was significantly reduced, and the high dose of HSE had the most substantial inhibitory effect, indicating that HSE could inhibit the promotion of M1 macrophages to secrete proinflammatory factors by fibroblasts.

In addition to inflammatory factors, fibroblasts can also secrete matrix metalloproteinases (MMPs) and matrix metalloproteinase inhibitors (TIMPs). MMPs, a Zn^2+^-dependent metalloproteinase superfamily, can degrade extracellular matrix components and participate in various processes of wound healing [25]. As a specific inhibitor of MMPs, TIMPs regulates its activity by regulating the level of MMPs and can bind and reversibly block the activity of MMPs [26]. It was found that the increase of MMP-2 in the exudate of chronic skin ulcers significantly affected the degradation of collagen and tissue destruction [27, 28]. MMP-3 participates in tissue remodeling, may degrade collagen fibrils, and activates other MMPs, such as MMP-1, MMP-7, and MMP-9, while TIMP-1 can promote wound healing. In this study, after coculture with M1 macrophages, fibroblasts that secreted MMP-1, MMP-2, and MMP-3 increased and that secreted TIMP-1 decreased, and after HSE intervention, fibroblasts that secreted MMP-1, MMP-2, and MMP-3 decrease, and TIMP-1 secretion increases, especially the high-dose HSE has the most potent regulation effect. Therefore, HSE can regulate the effect of M1 macrophages on the secretion of MMPs and TIMPs from fibroblasts. Fibroblast extracellular matrix deposition is an essential process of wound healing. In this process, the collagen produced is mainly type I collagen (COL1A1) and type III collagen (COL3A1). After coculture with M1 macrophages, fibroblasts secreted less COL1A1 and COL3A1, and after HSE intervention, fibroblasts secreted more COL1A1 and COL3A1 genes, indicating that HSE has a specific regulatory effect on the decreased secretion of COL1A1 and COL3A1 by M1 macrophages.

TGF-*β*1 is a beneficial fibroblast-causing factor related to human and other animal fibrosis [29]. TGF-*β*1 inhibits the degradation of ECM by blocking the secretion of proteases (MMPs) or stimulating the production of TIMPs [30]. Smad3 has been proven to be the profibrotic effect factor of TGF-*β*1; *α*-SMA is a specific marker of myofibroblasts [31]. The TGF-*β*1/Smad3 signaling pathway phosphorylates Smad3, transports it to the nucleus, and induces the expression of *α*-SMA in the nucleus, ultimately promoting cell fibrosis [32]. We detected the expression of *α*-SMA after different concentrations of HSE, intervened HSF, and initially proved that HSE could promote the expression of *α*-SMA of HSF, which means that HSE can promote cell fibrosis and is positively correlated with its concentration. To further explore the signaling pathway mechanism of HSE to promote cell fibrosis, we detected TGF-*β*1/Smad3 signaling pathway-related proteins, and the results demonstrated that after coculture with M1 macrophage supernatant, Smad3 phosphorylation was significantly reduced. However, the inhibition of Smad3 phosphorylation by the supernatant of M1 macrophages after HSE intervention was changed, and this change was not related to the concentration of HSE in M1 macrophages. The Smad3 phosphorylated protein expression of M1+HSE-H, M1+HSE-M, and M1+HSE-L groups was increased; at the same time, other TGF-*β*1/Smad3 pathway-related proteins such as c-Fos and c -Jun changes were similar to phosphorylated Smad3 expression (Figures 6(c) and 6(d)). These results indicate that M1 macrophages inhibit the HSF fibrosis process by inhibiting the TGF-*β*1/Smad3 signaling pathway, but HSE can restore the inhibition of the TGF-*β*1/Smad3 signaling pathway by M1 macrophages to promote HSF fibrosis process.

## 5. Conclusions

In summary, the intervention of Chinese herbal medicine preparation of HSE can regulate the effect of M1 macrophages on fibroblasts, promote the proliferation of fibroblasts, inhibit the secretion of proinflammatory factors and matrix metalloproteinases by fibroblasts, promote the secretion of TIMP-1, COL1A1, and COL3A1, and restore the inhibition of the TGF-*β*1/Smad3 signaling pathway, which mean HSE can be used as a possible external extract for the treatment of the chronic nonhealing wound. We will investigate whether the regulation of HSE on M1 macrophages will affect other cells, such as neutrophils and endothelial cells to provide more theoretical support for optimizing the preparation process of Huiyang Shengji ointment.

## Figures and Tables

**Table 1:** Overview of primers used for qRT-PCR analysis.

Target gene	Direction	Sequence (5′-3′)	Amplicon size (bp)
CCL2	Forward	ATAGGAAGATCTCAGTGCAGAGGCTC	175
	Reverse	TTCGGAGTTTGGGTTTGCTTGT	
CCL7	Forward	TCAATAAGAAAATCCCTAAGCAGAG	142
	Reverse	AAAGTCCTGGACCCACTTCTGT	
IL-6	Forward	AAAGATGGCTGAAAAAGATGGATG	88
	Reverse	CAAACTCCAAAAGACCAGTGATGAT	
MMP-1	Forward	ACGATTCGGGGAGAAGTGAT	120
	Reverse	AAGCCCATTTGGCAGTTGTG	
MMP-2	Forward	GAATGCCATCCCCGATAACCT	116
	Reverse	TTCACGCTCTTCAGACTTTGGTTC	
MMP-3	Forward	CTGAAGACTTTCCAGGGATTGACT	118
	Reverse	TGTCACTTTCTTTGCATTTGGGT	
MMP-4	Forward	GGTGCTGAAGGCAGGGACTC	136
	Reverse	CAGTGGCATCTGAGGGAAAGG	
TIMP-1	Forward	TACACTGTTGGCTGTGAGGAATG	140
	Reverse	CAAGGTGACGGGACTGGAAG	
C0L1A1	Forward	ACAAGAGGCATGTCTGGTTCG	237
	Reverse	CGGATCTCGATCTCGTTGGA	
COL3A1	Forward	CTACACAGTTCTGGAGGATGGTTGC	203
	Reverse	TTTTGTTGGGATTTCAGATAGAGTTT	
18srRNA	Forward	GTAACCCGTTGAACCCCATT	151
	Reverse	CCATCCAATCGGTAGTAGCG	

## Data Availability

The datasets used and/or analyzed to support the findings of this study are available from the corresponding author upon request.
